# Differences in tongue microbiota of older adults with a high fungal load according to tongue coating status

**DOI:** 10.1128/spectrum.01488-26

**Published:** 2026-09-21

**Authors:** Seijun Ganaha, Mikari Asakawa, Michiko Furuta, Shinya Kageyama, Jiale Ma, Rina Itazu, Eri Namoto, Yoshihisa Yamashita, Toru Takeshita

**Affiliations:** 1 Section of Preventive and Public Health Dentistry, Division of Oral Health, Growth and Development, Faculty of Dental Science, Kyushu University 594058, Fukuoka, Japan; 2 Section of Orthodontics and Dentofacial Orthopedics, Division of Oral Health, Growth and Development, Faculty of Dental Science, Kyushu University 594058, Fukuoka, Japan; Sichuan University, Chengdu, China

**Keywords:** older adults, ITS, 16S rRNA, tongue microbiota

## Abstract

The tongue microbiota of older adults requires careful attention because it can be aspirated into the lower respiratory tract due to impaired swallowing function. This study focused on differences in the tongue microbiota of older adults with a high fungal load, with and without tongue coating. We initially quantified the total fungal load of tongue microbiota in 145 individuals receiving commuting rehabilitation services (mean age, 87.6 ± 6.5 years) using quantitative polymerase chain reaction targeting the fungal ribosomal internal transcribed spacer (ITS) region. The fungal and bacterial compositions of 58 individuals with high fungal loads were determined based on the fungal ITS region and bacterial 16S rRNA gene amplicon sequencing, respectively. No significant differences were observed in the fungal load and composition of the tongue microbiota between individuals with no/slight and substantial tongue coating (Winkel Tongue Coating Index ≤3 and >3, respectively), and *Candida albicans* was the predominant fungal species of the tongue microbiota regardless of the tongue coating status. In contrast, individuals with no/slight tongue coating exhibited significantly lower bacterial loads and higher relative abundances of acidogenic taxa, including *Streptococcus mutans* and *Lactobacillus gasseri*, compared with those with substantial tongue coating. These bacterial communities were also distinct from those of individuals with no/slight tongue coating and a low fungal load. These findings indicate that the reduction in tongue coating accompanied by fungal overgrowth in older adults reflects alterations in the bacterial community, rather than differences in fungal composition.

**IMPORTANCE:**

Tongue microbiota status requires particular attention, especially among older adults, as microbiota can be aspirated into the lower respiratory tract due to impaired swallowing function, leading to pulmonary infections. Bacterial overgrowth on the tongue dorsum manifests as an accumulation of tongue coating, which serves as an indicator of tongue hygiene. However, an increase in fungal components in older adults is occasionally accompanied by a loss of tongue coating unrelated to good tongue hygiene, thereby complicating visual assessments. This study demonstrated that loss of tongue coating along with fungal overgrowth reflects the coincidence of alterations in the bacterial community and an increased prevalence of decayed teeth, indicating a higher risk of dental caries. The study findings highlight the importance of assessing both dental health status and tongue microbiota in older adults with slight tongue coating.

## INTRODUCTION

Oral cavity is densely colonized by diverse indigenous microorganisms that constitute different microbial communities in various ecological niches on intraoral surfaces. Two major oral diseases, dental caries and periodontitis, are primarily caused by the dental plaque microbiota formed on the tooth surface. The tongue microbiota serves as a primary source of oral microbial populations that are continuously swallowed with saliva (1–3). This microbiota requires particular attention, particularly in older adults, as it may be aspirated into the lower respiratory tract owing to impaired swallowing function and reduced coughing ability, leading to pulmonary infections (4). In addition, older adults are prone to the accumulation of tongue coatings because of reduced salivary flow and decreased tongue mobility (5, 6). Monitoring tongue microbiota status is important for maintaining systemic health in older adults.

The dorsum of the tongue has a large surface area with a papillary structure that can retain numerous microorganisms along with desquamated epithelial cells and oral debris (7, 8). Poor tongue hygiene generally leads to bacterial overgrowth, resulting in a visible coating of the tongue (9). Such coating serves as an indicator of hygiene status, and its mechanical removal can reduce bacterial load (10–13). However, in older adults, the absence of tongue coating does not necessarily indicate good tongue hygiene, as increased fungal colonization may occur even in the absence of visible tongue coating. In particular, the disappearance of tongue coating accompanied by lingual papillae atrophy is commonly diagnosed as erythematous and atrophic oral candidiasis (14, 15). In pseudomembranous candidiasis, another well-known form of oral candidiasis, the erythematous mucosal surface is covered by a confluent white plaque that can be gently wiped off (16). Fungal proliferation within the tongue microbiota complicates the visual assessment of tongue hygiene in older adults. Furthermore, information about variations in fungal-rich tongue microbiota according to tongue coating accumulation status is scant.

*Candida* species, including *C. albicans*, are widely recognized as the primary causative agents of oral candidiasis, including both erythematous and atrophic types (16–18). They are common commensal fungi present in healthy individuals. Loss of tongue coating may depend on interactions with diverse fungal and bacterial commensals that inhabit the microbiota. This study investigated the differences in the tongue microbiota associated with fungal overgrowth between individuals with and without tongue coating. Tongue microbiota samples were collected from older adults receiving commuting rehabilitation services, and individuals with high fungal loads in their tongue microbiota were selected. The fungal composition of the tongue microbiota was determined using fungal ribosomal internal transcribed spacer (ITS) region sequencing analysis and was compared between individuals with no/slight and substantial tongue coating. We also focused on the differences in bacterial composition as determined by 16S rRNA gene sequencing.

## MATERIALS AND METHODS

### Study population

This cross-sectional study included older adults receiving commuting rehabilitation services under Japan’s long-term care insurance system. From November 2023 to December 2023, we examined 171 individuals aged 65 years and older who attended two centers located in Aso City and Oguni Town, Kumamoto Prefecture, Japan. We excluded 26 individuals who refused to participate (*n* = 22) or used antibiotics (*n* = 4). A total of 145 individuals (43 men and 102 women) with a mean age of 87.6 ± 6.5 years were analyzed in this study. Written informed consent was obtained from all the participants. This study was approved by the Ethics Committee of Kyushu University (approval number 23334).

### Clinical information and tongue microbiota collection

Trained dentists conducted dental examinations. The number of present and decayed teeth and denture wearers were examined. Periodontal conditions were evaluated based on the periodontal pocket depth and bleeding on probing at the mid-buccal site of all teeth, excluding the third molars. The oral hygiene status was assessed based on the dental plaque score according to the Simplified Oral Hygiene Index (19). Images of the dorsal surface of the tongue were captured for each participant using an EyeSpecial C-IV (SHOFU Inc., Kyoto, Japan). Tongue coating was visually scored by summing the scores from six sections using a 0–2 score (0 = no coating, 1 = light coating, and 2 = severe coating), according to the Winkel Tongue Coating Index (WTCI) (20). Four examiners assessed the tongue coating adhesion and calculated the mean WTCI score. The tongue coating samples were collected using a sampling device based on a modified Braun Oral-B Pro 500 electric toothbrush (Procter and Gamble, Cincinnati, OH) (9). A circular bounded-fiber fabric (15 mm in diameter; Kao Corporation, Tokyo, Japan) attached to the brush head was placed at the center of the tongue dorsum, and vibrations were applied for 3 s to collect tongue-coated samples on the fabric. The fabric peeled from the brush head was immersed in 200 μL of lysis buffer containing 9 mM Tris-HCl, 0.9 mM EDTA, and 0.1% sodium dodecyl sulfate, and stored at –80°C until further analysis. DNA extraction from tongue coating samples was performed using a bead-beating method with 0.3 g zirconia–silica beads (0.1 mm diameter [Qiagen, Hilden, Germany]), as described previously (21). Body weight and height were measured, and body mass index (BMI; kg/m^2^) was calculated. Basic activities of daily living were evaluated using the Barthel Index (22). Cognitive function was assessed using the Mini-Mental State Examination (MMSE) (23), with scores ranging from 0 to 30 and higher scores indicating better cognition. The degree of oral moisture was evaluated using the modified Schirmer test, which is based on the Schirmer tear test routinely used by ophthalmologists to measure tear film wetness (24). During the measurement, the subjects were asked to position their tongue as far away from the filter paper strip as possible so that the test strip would not touch their tongue. A Schirmer Tear Production Measuring Strip (AYUMI Pharmaceutical Corporation, Tokyo, Japan) was held vertically using dental tweezers, and the rounded end was placed under the tongue for 1 min. Low oral moisture was defined as a color shift <8 mm (25).

### Quantitative polymerase chain reaction analysis

Quantitative polymerase chain reaction (PCR) analysis of the total fungal or bacterial density in the collected samples was performed using the KAPA SYBR Fast qPCR kit (Kapa Biosystems, Wilmington, MA, USA), according to the manufacturer’s instructions. Total fungal density was estimated using the primers 5.8SR (5′-TCG ATG AAG AAC GCA GC-3′) and ITS4 (5′-TCC TCC GCT TAT TGA TAT GC-3′), targeting the fungal ITS2 region of the rRNA gene. The ITS region of *C. albicans* was cloned into the pBluescript SK II (+) vector (Stratagene, La Jolla, CA, USA) and used as a real-time PCR control. Total bacterial density was estimated using the primers 806F (5′-TTA GAT ACC CYG GTA GTC C-3′) and 926R (5′-CCG TCA ATT YCT TTG AGT TT-3′), which amplify the V5 regions of the 16S rRNA gene. The 16S rRNA gene of *Porphyromonas pasteri* was cloned into the pBluescript SK II (+) vector (Stratagene) and used as a real-time PCR control. Real-time PCR was performed under the following cycling conditions: 95°C for 3 min, followed by 40 cycles of denaturation at 95°C for 3 s and annealing at 60°C for 20 s.

### Fungal ITS gene sequencing

The fungal ITS2 gene sequencing analysis was performed using tongue coating samples collected from 58 individuals with fungal density of ≥5,000 copies per sample (≥100 copies/μL of template DNA), as sufficient ITS region amplicons for sequencing analysis could be stably obtained from these samples (26). The ITS2 region of the rRNA gene from each sample was amplified using the following primers: 5.8 SR with Ion Torrent adapter A and a sample-specific 8-base tag sequence, and ITS4 with the Ion Torrent trp1 adapter sequence. PCR amplification, purification, and quantification of each adaptor were performed as previously described (26). Emulsion PCR and enrichment of template-positive particles were performed using Ion 520 and Ion 530 Kit-OT2 (Thermo Fisher Scientific, Waltham, MA, USA) with Ion One Touch 2 system (Thermo Fisher Scientific). The enriched particles were loaded onto an Ion 530 Chip (Thermo Fisher Scientific), and sequencing was performed on an Ion GeneStudio S5 System (Thermo Fisher Scientific) using the Ion 520 and Ion 530 Kit-OT2 (Thermo Fisher Scientific).

### Bacterial 16S rRNA gene sequencing analysis

Bacterial 16S rRNA gene sequencing was performed using tongue coating samples collected from 74 individuals with a high fungal load and a no/slight tongue coating with a low fungal load. The V1–V2 region of the 16S rRNA gene from each sample was amplified using the following primers: 8F (5′-AGA GTT TGA TYM TGG CTC AG-3′) with Ion Torrent adaptor A and the sample-specific 8-base tag sequence, and 338R (5'- TGC CTC CCG TAG GAG T-3′) with Ion Torrent trP1 adaptor sequence. PCR amplification, purification, and quantification of each adaptor were performed as previously described (27). Emulsion PCR and enrichment of template-positive particles were performed using Ion 520 and Ion 530 Kit-OT2 (Thermo Fisher Scientific) with an Ion One Touch 2 system (Thermo Fisher Scientific). The enriched particles were loaded onto an Ion 530 Chip (Thermo Fisher Scientific), and sequencing was performed on an Ion GeneStudio S5 System (Thermo Fisher Scientific) using the Ion 520 and Ion 530 Kit-OT2 (Thermo Fisher Scientific).

### Data analysis and taxonomy assignment

Raw sequence reads were quality filtered using a script written in R (version 4.2.1). The reads were excluded from the analysis when they exhibited ≤200 bases or did not include the correct forward and reverse primer sequences. The remaining reads were demultiplexed by examining the 8-base tag sequence, and the forward and reverse primer sequences were trimmed. Quality-checked reads were further processed using the *dada2* package (version 1.32.0) in R, including quality filtering, denoising, and chimera filtering procedures with settings for Ion Torrent reads, and an amplicon sequence variant (ASV) table was produced (28). ASVs observed in the negative control were excluded from the ASV table and subsequently analyzed as PCR contaminants. The taxonomy of each sequence was determined at the species level using BLAST (29) against 17,495 fungal ITS region sequences in UNITE (version 9.0) (30) and 1,015 oral bacterial 16S rRNA gene sequences (16S rRNA RefSeq version 15.22) in the expanded Human Oral Microbiome Database (eHOMD) (31). Nearest-neighbor species with ≥98% identity in fungal species or with ≥98.5% identity in bacterial species were selected as candidates for each sequence. The genus-level taxonomy of each sequence variant was determined using the RDP classifier with a minimum support threshold of 80% and RDP taxonomic nomenclature (32). We constructed a species-level table from the ASV table by clustering ASVs assigned to the same reference sequences in UNITE or eHOMD. ASVs without hits against UNITE were excluded from fungal analysis. The Shannon index and bacterial phylogenetic diversity per sample were calculated following rarefaction at a depth of 2,000 and 4,000 reads for fungal and bacterial diversity, respectively, using R software.

### Statistical analysis

All statistical analyses were performed using R software (version 4.2.1). Differences in microbiota-related factors (total fungal and bacterial densities and alpha diversity indices) and health status between individuals with no/slight and substantial tongue coating and a high fungal load were determined using Wilcoxon rank-sum and Fisher’s exact tests. The dissimilarity in fungal and bacterial compositions among the samples was evaluated using the Aitchison distance, based on centered log-ratio-transformed abundance data at the species level (33). Permutational multivariate analysis of variance (PERMANOVA) based on the Aitchison distance was used to assess the correlation between tongue microbiota composition and each profile, using the Adonis function in the *vegan* library (version 2.7.3). Differentially abundant taxa between individuals with no/slight and substantial tongue coating and a high fungal load were identified using MaAsLin2 (Microbiome Multivariable Associations with Linear Models; *Maaslin2* package version 1.18.0) based on a centered log-ratio (CLR) transformed genus- and species-level tables (34). Features with a prevalence <10% were excluded during model fitting in MaAsLin2. Features with a Benjamini-Hochberg-corrected *q*-value of <0.25 (default setting) were considered significant. We analyzed a co-occurrence network among 84 bacterial and 18 fungal species with a prevalence of ≥20% in the tongue microbiota of 56 individuals with a high fungal load using FastSpar (version 1.0.0), a rapid C++ implementation of the SparCC algorithm (35). Correlations were considered statistically significant at *P* < 0.05 and an absolute correlation coefficient (*r*) > 0.3. These correlations were visualized using the *sna* package (version 2.8). Estimated absolute abundance of predominant fungal species (prevalence ≥10%) was calculated by multiplying the relative abundance of the predominant fungal species by the total fungal load of the tongue microbiota, adding a pseudo-count, and applying a log transformation (36).

## RESULTS

### Selection of older participants with a high fungal load and their tongue coating status

**Fig 1:**
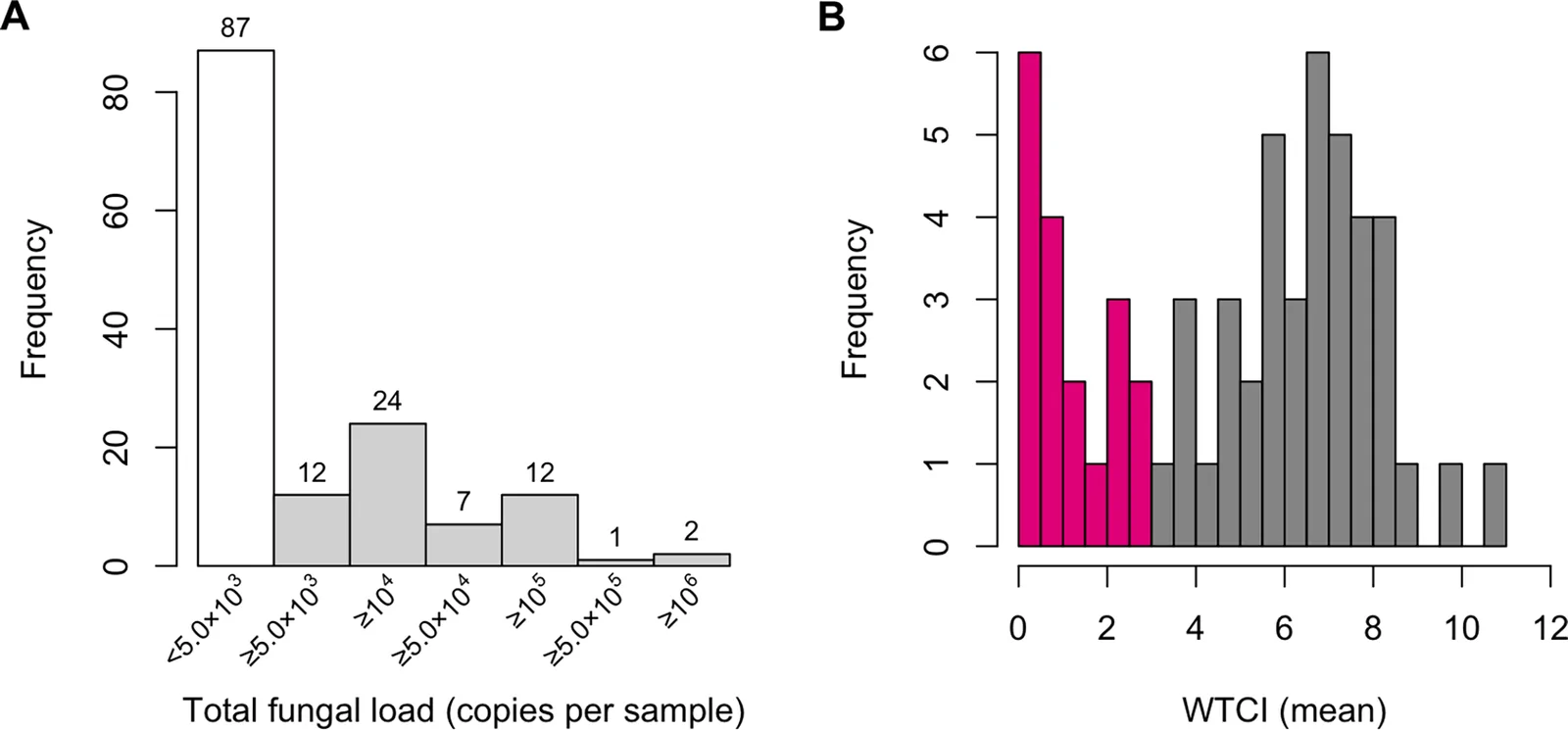
Distributions of total fungal load in the tongue microbiota and WTCI. (**A**) Histogram of total fungal load in the tongue microbiota of 145 individuals. (**B**) Histogram of mean WTCI score of 58 individuals with a high fungal load (≥5,000 copies). The pink bars indicate the distribution of individuals with no/slight tongue coating, whereas the dark gray bars indicate the distribution of individuals with substantial tongue coating.

**Fig 2:**
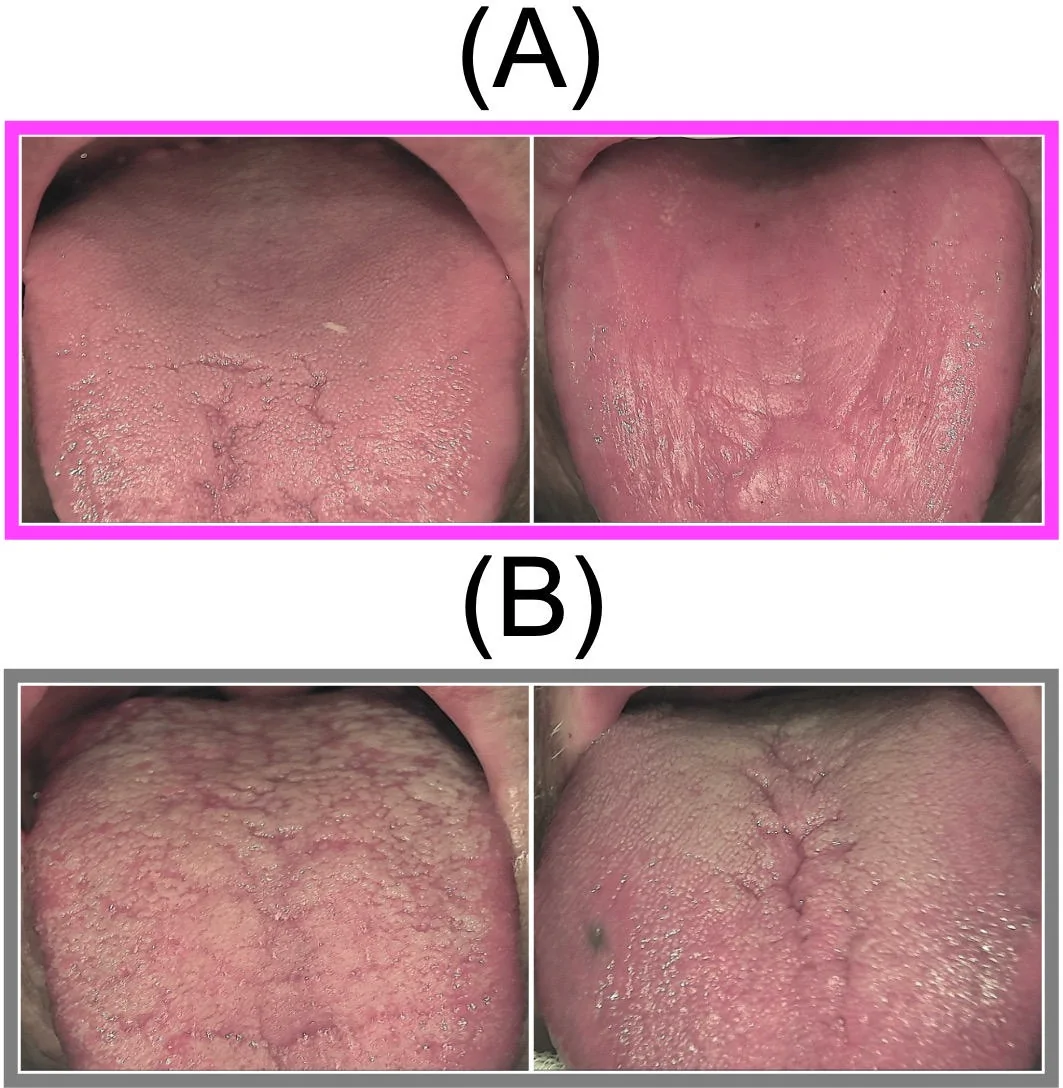
Tongue coating images of individuals with a high fungal load. (**A**) Tongue coating images of individuals with no/slight tongue coating. (**B**) Tongue coating images of individuals with substantial tongue coating.

This study initially determined the total fungal load in the tongue microbiota of 145 older participants using quantitative PCR analysis targeting the fungal ribosomal ITS2 region. Fifty-eight participants (40.0%) demonstrated a total fungal load exceeding 5,000 copies and were classified as having a high fungal load in this study (Fig. 1A). We assessed the tongue coating status based on the mean WTCI score evaluated by four examiners. The WTCI scores in individuals with a high fungal load exhibited a bimodal distribution, and we categorized the individuals into two groups with no/slight (≤3) and substantial (>3) tongue coating (Fig. 1B and 2). No individual presented with obvious confluent white patches on the tongue, which is a characteristic finding of oral thrush.

### Tongue coating status and oral and general health conditions

We compared oral and general health conditions in 58 individuals with a high fungal load according to the tongue coating accumulation status. No significant differences were observed in age, periodontal condition, denture wearing, and degree of oral moisture among individuals with no/slight and substantial tongue coating (Table 1). In contrast, individuals with no/slight tongue coating exhibited significantly more decayed teeth and lower Barthel index scores, indicating reduced physical function, than those with a substantial tongue coating (Table 1; Table S1).

**TABLE 1:** Characteristics of individuals with no/slight and substantial tongue coating and a high fungal load^*a*^

Characteristics	No/slight (*n* = 18)	Substantial (*n* = 40)	*P* value
Age (mean ± SD)	89.3 ± 6.4	88.3 ± 5.4	0.316
Female (*n*, %)	13 (72.2)	28 (70.0)	1
BMI (mean ± SD)	21.7 ± 3.9	22.0 ± 4.0	0.617
Barthel index (mean ± SD)	77.1 ± 15.9	85.6 ± 14.1	0.046
MMSE (mean ± SD)	18.8 ± 6.4	21.8 ± 5.2	0.130
Number of present teeth (mean ± SD)	11.6 ± 8.8	11.2 ± 8.7	0.893
Edentulous (*n*, %)	4 (22.2)	5 (12.5)	0.438
Dentulous with ≥3 decayed teeth (*n*, %)	10 (71.4)	12 (34.3)	0.027
Number of decayed teeth (mean ± SD)	6.6 ± 5.1	2.3 ± 3.6	0.004
Probing pocket depth (mean ± SD)	3.3 ± 0.5	3.1 ± 1.0	0.056
Plaque score (mean ± SD)	2.0 ± 0.8	1.7 ± 0.9	0.470
Percentage of teeth with bleeding on probing (mean ± SD)	25.2 ± 21.8	25.4 ± 30.4	0.633
Denture users (*n*, %)	15 (83.3)	32 (80.0)	1
Low oral moisture (*n*, %)	6 (33.3)	12 (30.0)	1
Oral moisture (mean ± SD)	10.6 ± 3.6	10.5 ± 4.7	0.643
Regular dental check-up (*n*, %)	2 (11.1)	6 (15.0)	0.701

^*a*^ SD, standard deviation. The Wilcoxon rank-sum and Fisher’s exact tests were used for comparison. Of the 28 individuals, 9 edentulous older individuals were excluded based on the number of decayed teeth, mean probing pocket depth, mean plaque score, and percentage of teeth with bleeding on probing.

### Fungal composition of the tongue microbiota and tongue coating status

The fungal composition of the 58 individuals was determined using fungal ribosomal ITS2 region sequencing to investigate differences in fungal communities associated with tongue coating status. A total of 3,069,401 quality-passed fungal ITS2 gene sequences were obtained using Ion GeneStudio S5. Two individuals (one from each profile) were excluded from the fungal compositional analysis because of insufficient reads after quality filtering.

**Fig 3:**
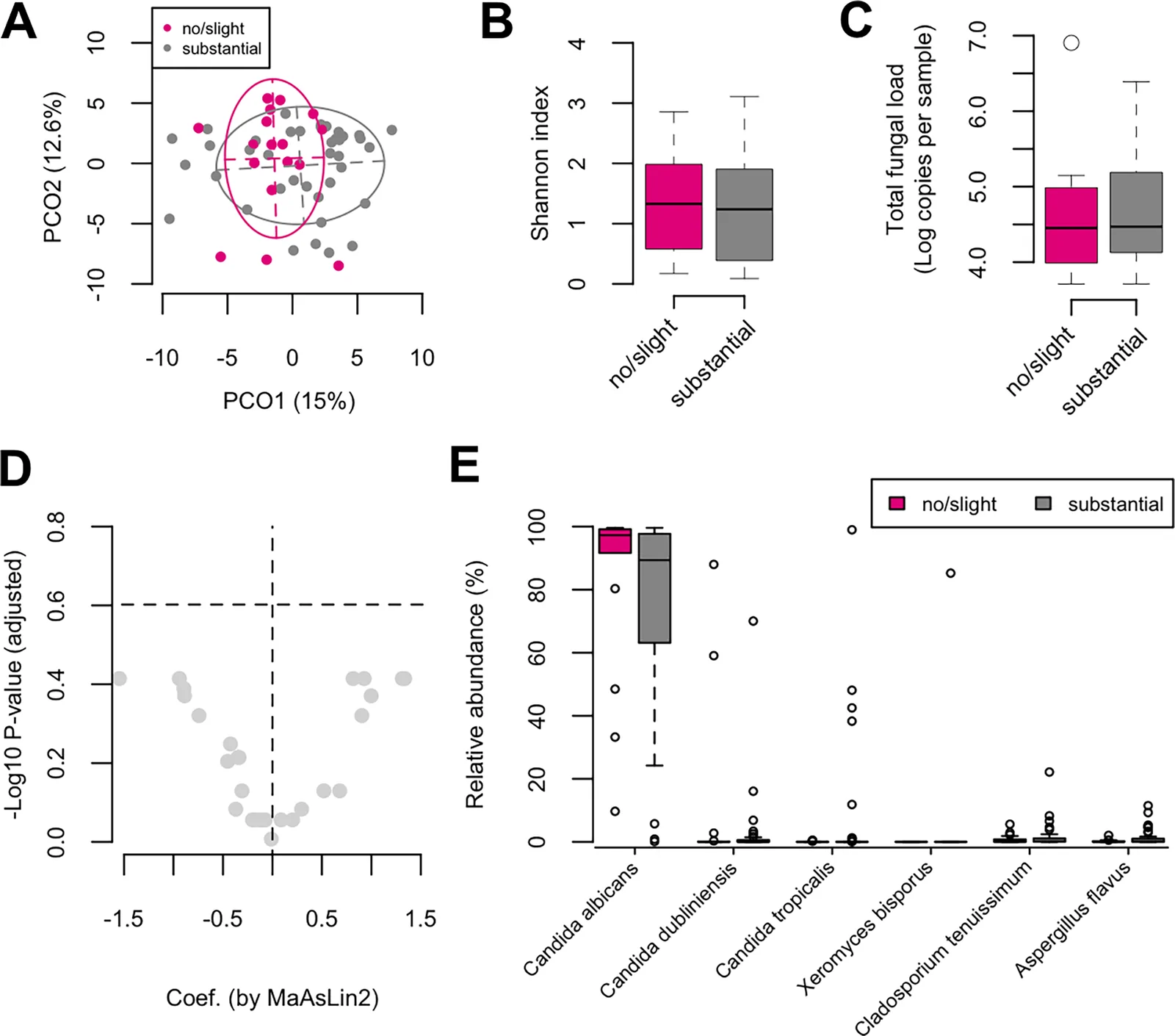
Comparison of tongue fungal communities between individuals with no/slight and substantial tongue coating, along with a high fungal load. (**A**) Principal coordinate analysis (PCoA) plot of fungal composition with a high fungal load based on Aitchison distance at the species level. Samples from individuals with no/slight and substantial tongue coating and a high fungal load are shown in different colors. Ellipses cover 67% of samples belonging to each profile. Statistical significance was assessed using PERMANOVA. (**B**) Shannon diversity index for each group. Statistical significance was assessed using the Wilcoxon rank-sum test. (**C**) Total fungal load in the tongue microbiota of each group. Statistical significance was assessed using the Wilcoxon rank-sum test. (**D**) Differentially abundant species between individuals with no/slight and substantial tongue coating and a high fungal load identified by MaAsLin2. Dots indicate fungal species with a prevalence of ≥10%. The *x*-axis shows effect size (coefficient), and the *y*-axis shows −log10 (adjusted *P* value). Threshold: adjusted *P* < 0.25. (**E**) Relative abundance of six predominant fungal species with a mean relative abundance of ≥1% in individuals with no/slight and substantial tongue coating and a high fungal load.

The tongue microbiota of all individuals contained *C. albicans* regardless of the amount of tongue coating (Fig. S1). Alpha and beta diversity indices of the tongue microbiota were not significantly different between individuals with no/slight and substantial tongue coating (Fig. 3A and B). No fungal species were identified as discriminant taxa between the two groups based on MaAsLin2 analysis (Fig. 3D and E). In addition, total fungal loads did not differ significantly between the two groups (*P* = 0.572, Wilcoxon rank-sum test; Fig. 3C).

### Bacterial communities of the tongue microbiota and tongue coating status

We subsequently compared bacterial communities of the tongue microbiota between individuals with no/slight and substantial tongue coating. The total bacterial load in the tongue microbiota was determined by quantitative PCR targeting the V5 region of the 16S rRNA gene. The bacterial composition was also determined based on the V1–V2 regions of the 16S rRNA gene sequencing using Ion GeneStudio S5, which yielded 1,096,483 quality-passed reads.

**Fig 4:**
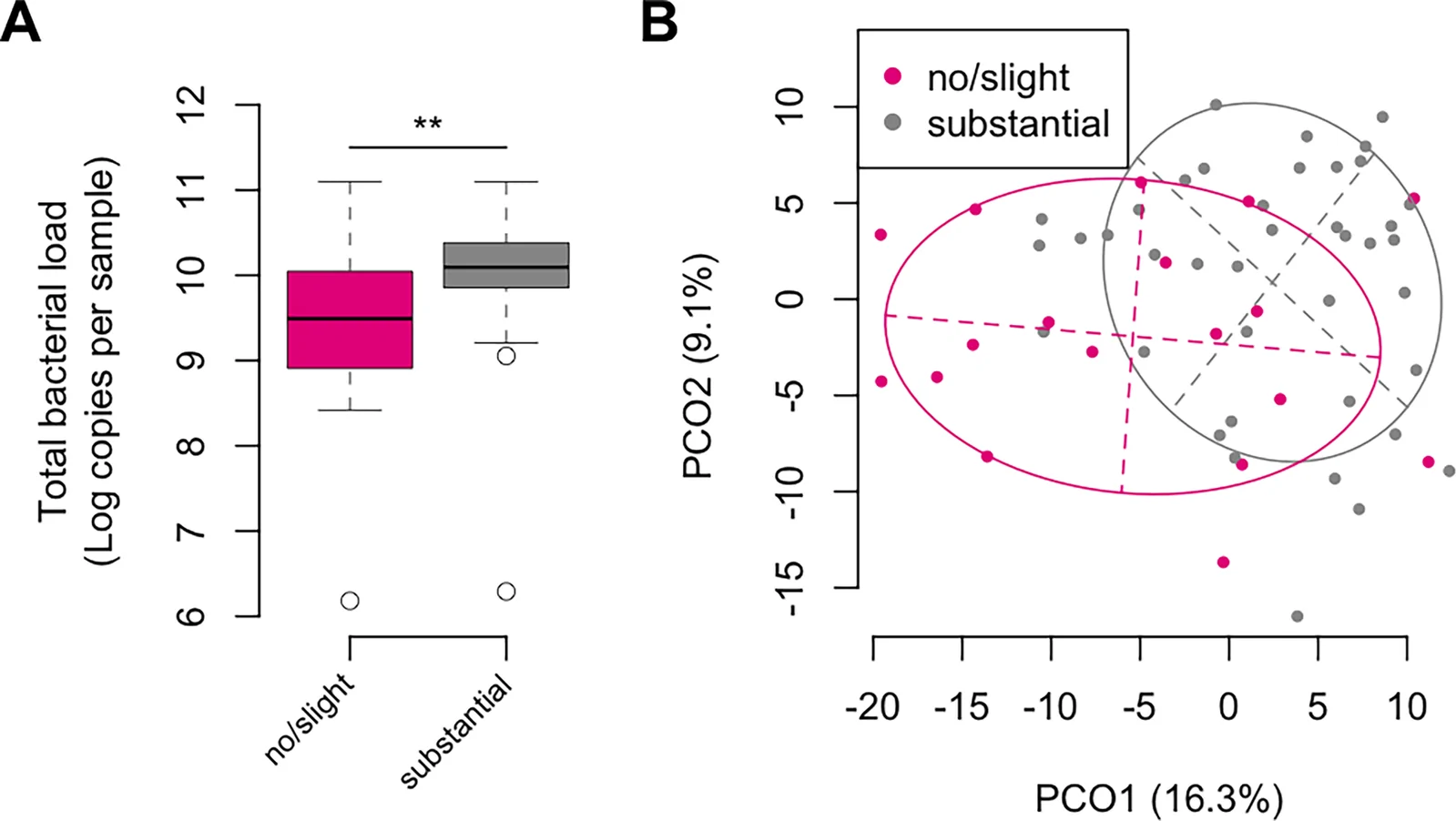
Comparison of tongue bacterial communities between individuals with no/slight and substantial tongue coating, along with a high fungal load. (**A**) Total bacterial load in the tongue microbiota of each group. Statistical significance was assessed using the Wilcoxon rank-sum test. ^**^P < 0.01. (**B**) PCoA plot of bacterial composition with a high fungal load based on Aitchison distance at the species level. Samples from individuals with no/slight and substantial tongue coating and a high fungal load are shown in different colors. Ellipses cover 67% of samples belonging to each profile. Statistical significance was assessed using PERMANOVA.

**Fig 5:**
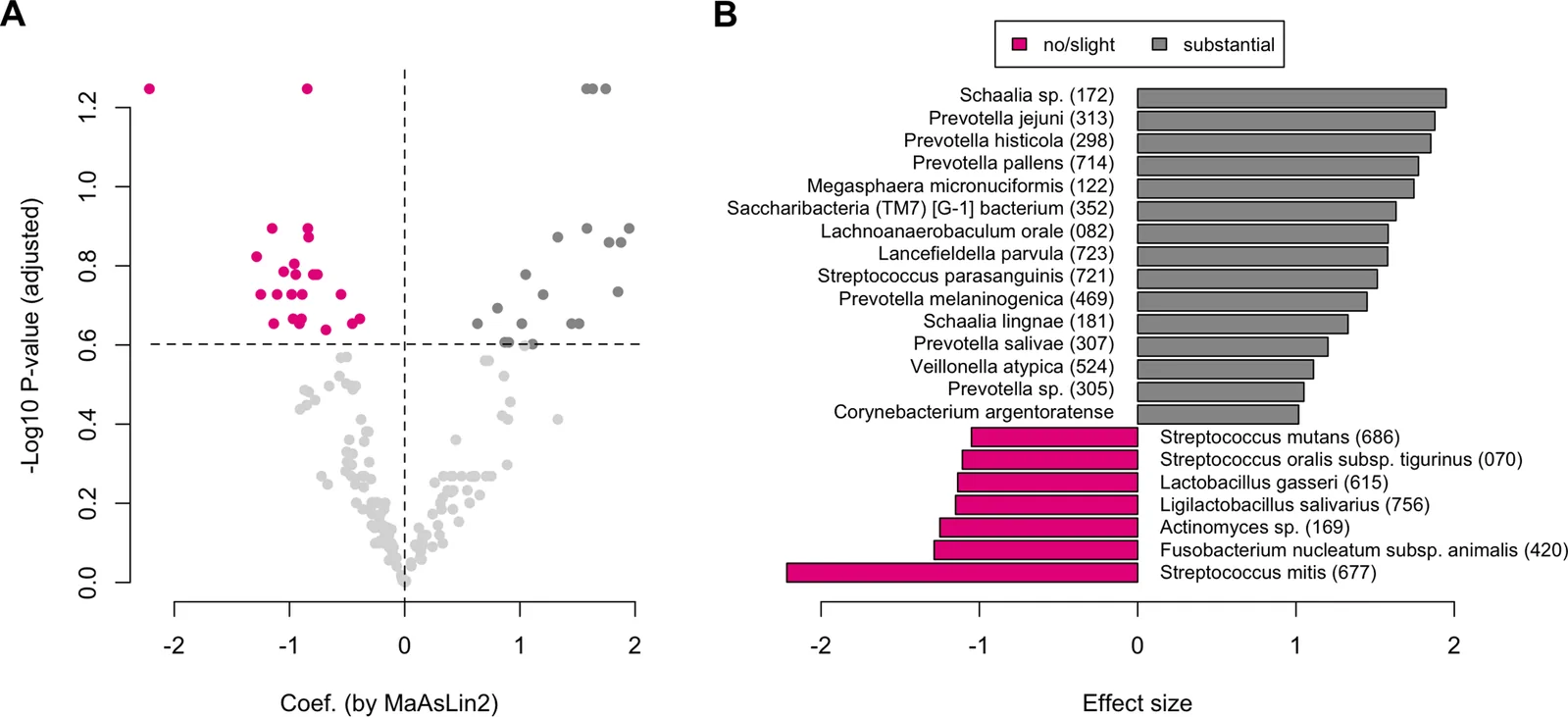
Differentially abundant bacterial taxa between individuals with no/slight and substantial tongue coating, along with a high fungal load. (**A**) Differentially abundant bacterial taxa were identified between individuals with no/slight and substantial tongue coating and a high fungal load using MaAsLin2. Dots indicate species with a prevalence of ≥10%. The x-axis shows the effect size (coefficient), and the y-axis represents −log10 (adjusted *P* value). Threshold: adjusted *P* < 0.25. Negative values indicate species more abundant in individuals with no/slight tongue coating (pink), and positive values indicate species more abundant in individuals with substantial tongue coating (dark gray). (**B**) Differentially abundant species between individuals with no/slight and substantial tongue coating. Bar plots show effect sizes from MaAsLin2 for each species. Species with *q* value < 0.25 and an absolute effect size >1.0 are shown. Human microbial taxon (HMT) numbers from the expanded Human Oral Microbiome Database (eHOMD) are provided in parentheses following bacterial names. Species without HMT numbers were assigned based on the SILVA database.

The total bacterial load of individuals with no/slight tongue coating was significantly lower than those with a substantial tongue coating (Fig. 4A). Although no significant differences were observed in the alpha diversity indices (Table 2), beta-diversity analysis based on Aitchison distance demonstrated that the overall bacterial composition was significantly different among individuals with no/slight and substantial tongue coating (*P* < 0.001, PERMANOVA; Fig. 4B). MaAsLin2 analysis revealed that bacterial species, including *Streptococcus mitis*, *Fusobacterium nucleatum* subsp. *animalis*, *Actinomyces* HMT-169, *Ligilactobacillus salivarius*, *Lactobacillus gasseri*, *Streptococcus oralis* subsp. *tigurinus*, and *Streptococcus mutans*, were differentially abundant in individuals with no/slight tongue coating. In contrast, bacterial taxa, including *Prevotella* species such as *Prevotella jejuni*, *Prevotella histicola*, *Prevotella pallens*, *Prevotella melaninogenica*, *Prevotella salivae*, and *Prevotella* HMT-305, were differentially abundant in individuals with a substantial tongue coating (Fig. 5; Fig. S2 and S3).

**TABLE 2:** Bacterial diversity in the tongue microbiota of individuals with no/slight and substantial tongue coating, along with a high fungal load^*a*^

Characteristics	No/slight (*n* = 18)	Substantial (*n* = 40)	*P* value
Bacterial diversity			
Shannon index (mean ± SD)	4.5 ± 0.8	4.7 ± 0.7	0.259
Phylogenetic diversity (mean ± SD)	13.2 ± 3.8	13.0 ± 3.7	0.901

^*a*^ SD, standard deviation. The Wilcoxon rank-sum test was used for comparison.

### Association between alteration of the bacterial community and fungal overgrowth

Next, the bacterial composition of individuals with no/slight tongue coating and a high fungal load was compared with that of individuals with a low fungal load (*n* = 16; Fig. S4). The overall bacterial composition in individuals with no/slight tongue coating differed significantly between the high and low fungal load groups (*P* < 0.001, PERMANOVA; Fig. S5). The discriminant taxa between the abovementioned two groups shown in Fig. 5 were not predominant in individuals with no/slight tongue coating and a low fungal load (Fig. S6). These results suggest that alterations in the bacterial community are accompanied by fungal overgrowth rather than by a small amount of tongue coating.

### Cross-kingdom co-occurrence networks in tongue microbiota

**Fig 6:**
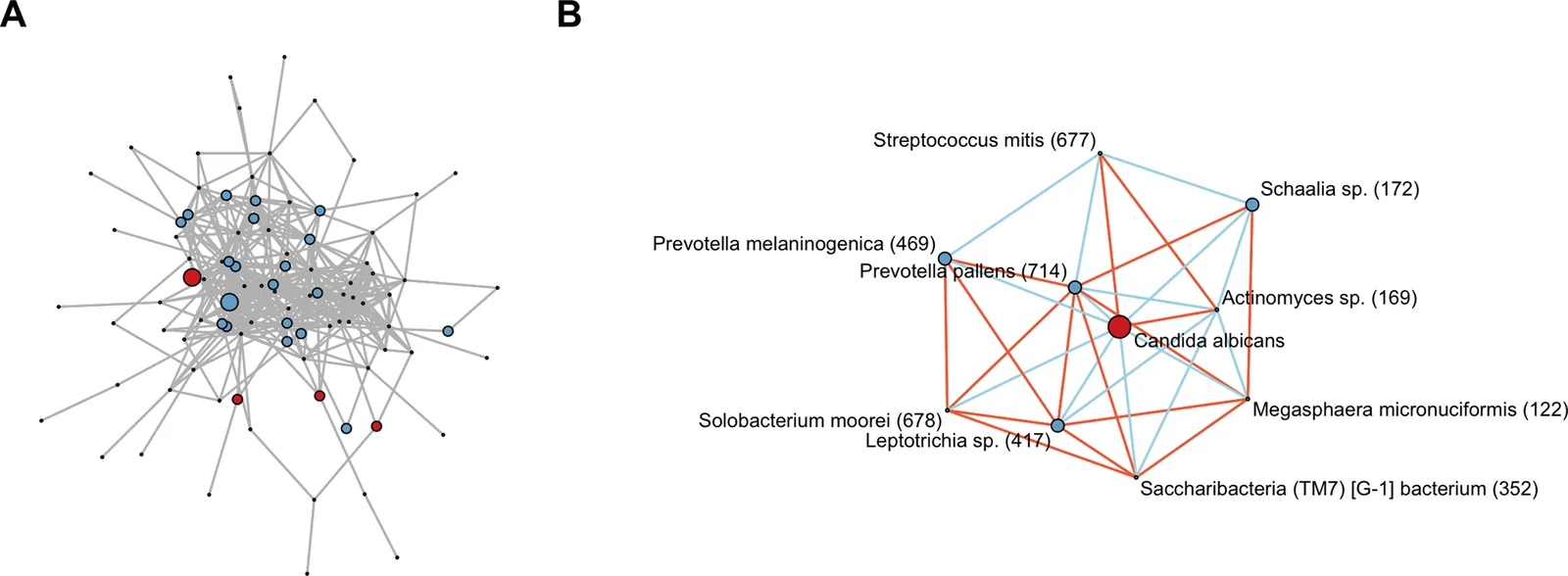
Cross-kingdom co-occurrence network in tongue microbiota. Co-occurrence relationships with FastSpar *R* ≥ 0.3 are visualized as edges. Blue and red nodes, respectively, indicate bacterial and fungal species in tongue microbiota. Node size indicates the mean relative abundance of each species. (**A**) Co-occurrence network of all taxa with ≥20% prevalence. (**B**) Co-occurrence network correlated with *C. albicans*. Nine bacterial species directly correlate with *C. albicans*. Orange and blue edges indicate positive and negative correlations. Human microbial taxon (HMT) numbers in the expanded Human Oral Microbiome Database (eHOMD) are shown in parentheses after bacterial names.

We analyzed a co-occurrence network of bacterial and fungal species with ≥20% prevalence in the tongue microbiota. The network comprised 93 species and 854 connections (edges; Fig. 6A). Among these, seven of the nine species that directly correlated with *C. albicans* corresponded to the discriminant bacterial species identified in Fig. 5 (Fig. 6B).

## DISCUSSION

This study revealed differences in the composition of the tongue microbiota in older individuals with a high fungal load with and without tongue coating. *C. albicans* was the primary fungal component of the tongue microbiota in both cases, and no significant differences were observed in the fungal load and composition (Fig. 3). Furthermore, no significant differences were observed when the estimated absolute abundance of predominant fungal species, calculated by multiplying their relative abundance by the total fungal load of the tongue microbiota, was used in the analysis (data not shown). In contrast, bacterial communities in individuals with no/slight tongue coating were significantly less dense (Fig. 4A) and exhibited higher relative abundances of *Streptococcus* and *Lactobacillus* species than those in individuals with substantial tongue coating (Fig. 5). However, this bacterial profile was not observed in individuals with no/slight tongue coating and a low fungal load, which could be regarded as a physiologically good tongue microbiota (Fig. S6) (37, 38). These findings suggest that the loss of tongue coating reflects the coincidence of alterations in the bacterial community with fungal overgrowth in the tongue microbiota.

Notably, the bacterial community in individuals with no/slight tongue coating and a high fungal load was characterized by enrichment of *Streptococcus* species, including *S. mitis*, *S. oralis* subsp. *tigurinus*, and *S. mutans* (Fig. 5). This finding is consistent with a previous shotgun metagenomic study demonstrating enrichment of *Streptococcus* species in the salivary microbiota of patients with chronic erythematous candidiasis (17). Together with our previous findings of increased *Streptococcus* and *Lactobacillus* abundances in individuals with a high fungal load (26), these results suggest that a *Streptococcus*-dominant community is associated with tongue coating loss accompanied by fungal overgrowth rather than with oral candidiasis in general.

Interactions between *Candida* and *Streptococcus* species might be influenced by pH, nutrients, and host factors, such as salivary components (39). For example, streptococcal species co-aggregate with *C. albicans*, particularly yeast, under glucose-starvation (40). In addition, coculturing *C. albicans* with streptococcal species in the presence of sucrose promotes *C. albicans* growth (41). The altered bacterial composition characterized by a predominance of *Streptococcus* in this study of older individuals with a high fungal load is consistent with previous findings (42, 43). Considering the potential interactions between *Candida* and Streptococcus species, our results are reasonable.

Notably, more decayed teeth were present in individuals with no/slight tongue coating than in those with a substantial tongue coating (Table 1). The bacterial communities in individuals with no/slight tongue coating were less dense but more predominantly cariogenic pathogens, such as *S. mutans* and *Lactobacillus* species, than those in individuals with a substantial tongue coating, implying more acidic conditions in their oral cavity. In addition, several studies on early childhood caries have demonstrated that co-infection with *C. albicans* and *S. mutans* enhances cariogenic virulence (44–48). Dysbiotic oral microbiota, including cross-kingdom interactions between bacterial and fungal components, would result in a higher prevalence of dental caries. Root caries is one of the most common public dental health concerns in older adults and a major cause of tooth loss (49–51). Our results suggest that regulation of fungal components in the microbiota might prevent alterations in the bacterial community, leading to suppression of the onset and progression of root caries.

Dry mouth is associated with a higher prevalence of oral *Candida* colonization and oral candidiasis, as well as a high risk of dental caries (52, 53). The salivary flow rate also affects intraoral pH levels (54), and saliva contains histatins, which function as antifungal peptides (55). However, no significant difference was observed in the oral moisture status between individuals with no/slight and substantial tongue coating (Table 1; Table S1). This suggests that alterations in the bacterial community associated with fungal overgrowth are not only due to oral dryness. However, careful attention is needed when interpreting these results because the degree of oral moisture was evaluated using the modified Schirmer’s test, which is a screening tool for older adults who have difficulty collecting saliva (24). Future studies based on a more accurate evaluation of oral dryness under normal conditions, such as measurement of resting salivary flow, are required to investigate the relationship between oral moisture and tongue coating status under conditions of fungal overgrowth.

Although denture use is a major predisposing factor for *Candida* overgrowth (42, 56), no significant difference in the number of denture wearers was observed across tongue coating status groups (Table 1; Table S1). The study population involved older adults receiving commuting rehabilitation services at daycare centers, many of whom used these services one to three times per week. Therefore, their denture hygiene status may have remained relatively clean. This study did not evaluate denture hygiene status. Further studies are required to evaluate the association between fungal communities and denture use, considering denture hygiene in older adults.

A significant decline in physical function was observed among individuals with no/slight tongue coating compared with those with a substantial tongue coating (Table 1). Our previous study indicated that the tongue microbiota with a high density of fungal population and predominance of *Streptococcus* and *Lactobacillus* were associated with lower physical function in older adults receiving community support and home care services (26). The possible mechanisms by which tongue microbiota with no/slight tongue coating and a high fungal load cause an adverse effect on physical function remain unclear. However, careful attention should be paid to the tongue coating status in older adults, considering the possibility that it contributes to worsening of the host’s health conditions.

Although *Candida* species are dimorphic fungi that grow in yeast and hyphal forms, this study did not clarify whether the growth forms of yeasts differ between light and severe tongue coatings. However, a retrospective study of tongue smears showed that the hyphal form of *Candida* was more frequent when the tongue dorsum appeared atrophic (57). Considering that streptococci use glycans that cover the hyphae for adhesion (58), a change in the growth form of *Candida* might contribute to a higher predominance of *Streptococcus* in individuals with no/slight tongue coating and a high fungal density. Future studies should evaluate *Candida* hyphae and focus on cross-kingdom interactions in tongue microbiota.

*Fusobacterium nucleatum* is an anaerobic bacterium primarily associated with the subgingival environment, and its prevalence in tongue coating and supragingival biofilms is generally considered low (59). In our study, *F. nucleatum* subsp. *animalis* was enriched in individuals with a no/slight tongue coating and a high fungal load (Fig. 5). These individuals tended to have deeper pockets than those with a substantial tongue coating (*P* = 0.056; Table 1), suggesting that the presence of *F. nucleatum* subsp. *animalis* in tongue coating may reflect microbial transfer from periodontal sites. However, the distribution of *F. nucleatum* subspecies across different oral niches remains poorly understood. Previous studies have shown that *F. nucleatum* subsp. *polymorphum* is associated with healthy dental plaque, whereas *F. nucleatum* subsp. *animalis* is enriched in inflammatory sites such as odontogenic abscesses (60). Our findings demonstrate the presence of *F. nucleatum* subsp. *animalis* in tongue coating and highlight the need for further subspecies-level studies to clarify its ecological distribution.

This study had a few limitations. First, there was no information regarding medications, except for antibiotics. Systemic corticosteroids, immunomodulatory drugs, and xerogenic drugs are known factors that contribute to oral candidiasis (16). As older adults often take multiple medications, future studies should consider the impact of these medications on the microbiota. Second, the present study had a small sample size. Further studies with larger sample sizes are needed to confirm these findings. Third, our findings cannot be generalized because our study population consisted of older adults. Fourth, we analyzed only those with high-density fungal communities to avoid misidentifying transient or contaminated taxa as oral commensals. Finally, the determining factor of whether fungal overgrowth coincides with alterations in the bacterial community remains unclear in this study. Transcriptomic and proteomic analyses targeting fungal components, including *C. albicans*, would help clarify the mechanism underlying dysbiotic shifts in the bacterial community.

The present study demonstrated that the microbial composition of the tongue microbiota in older adults with a high fungal load was associated with variations in tongue coating status. Bacterial communities in individuals with no/slight tongue coating and a high fungal load were less dense but more dominated by cariogenic taxa than those with a substantial tongue coating and a high fungal load. The increased prevalence of decayed teeth also implies a highly acidic oral environment in these individuals. Collectively, these findings highlight the importance of evaluating both microbiota composition and dental health status in older adults with a slight amount of tongue coating.

## Data Availability

The sequence data have been deposited in the DDBJ Sequence Read Archive under the accession number PRJDB42854.

## SUPPLEMENTAL MATERIAL

Supplemental figures and table spectrum.01488-26-s0001.docx Fig. S1 to S6 and Table S1.

ASM does not own the copyrights to Supplemental Material that may be linked to, or accessed through, an article. The authors have granted ASM a non-exclusive, world-wide license to publish the Supplemental Material files. Please contact the corresponding author directly for reuse.
