# Supplementary material for: Differences in tongue microbiota of older adults with a high fungal load according to tongue coating status
Source: Microbiol Spectr. 2026 Sep 21;14(11):e01488-26. doi: 10.1128/spectrum.01488-26 (PMC13729161; doi:10.1128/spectrum.01488-26)
Supplement: Supplementary file 1 [file spectrum.01488-26-s0001.docx]

Supplementary Appendix

**Differences in tongue microbiota of older adults with a high fungal load according to tongue coating status**

Seijun Ganaha^a†^, Mikari Asakawa^a†^, Michiko Furuta^a^, Shinya Kageyama^a^, Jiale Ma^a^,　Rina Itazu^a, b^, Eri Namoto^a^, Yoshihisa Yamashita^a^, Toru Takeshita^a^*

Section of Preventive and Public Health Dentistry, Division of Oral Health, Growth and Development, Faculty of Dental Science, Kyushu University, Fukuoka, Japan^a^; Section of Orthodontics and Dentofacial Orthopedics, Division of Oral Health, Growth and Development, Faculty of Dental Science, Kyushu University, Fukuoka, Japan^b^

*Correspondence to: Toru Takeshita, [taketooo@dent.kyushu-u.ac.jp](mailto:taketooo@dent.kyushu-u.ac.jp)

^†^These authors contributed equally to this work.

Contents

Fig. S1. Relative and estimated absolute abundances of predominant fungal taxa in individuals with no/slight and substantial tongue coating with a high fungal load.

Fig. S2. Differentially abundant bacterial genera in individuals with no/slight and substantial tongue coating with a high fungal load.

Fig. S3. Relative abundance and detection ratio of differentially abundant bacterial taxa between individuals with no/slight and substantial tongue coating with a high fungal load.

Fig. S4. Representative tongue coating images of individuals with no/slight tongue coating and a low fungal load.

Fig. S5. Principal coordinate analysis (PCoA) of bacterial composition among individuals classified by tongue coating status and fungal load.

Fig. S6. Relative abundances of seven discriminant bacterial species identified in Figure 5 in individuals with no/slight tongue coating and a high fungal load and in the other three groups.

Table S1. Characteristics of individuals classified according to tongue coating status and fungal load.

**Fig. S1. Relative and estimated absolute abundances of predominant fungal taxa in individuals with no/slight and substantial tongue coating with a high fungal load.**

Fungal species with ≥ 10% of max relative abundance in individuals with no/slight and substantial tongue coating and a high fungal load. (A) Relative abundances of predominant fungal species. (B) Estimated absolute abundances of predominant fungal species. Estimated absolute abundance was calculated by multiplying the relative abundance of the predominant fungal species by total fungal load of the tongue microbiota, adding a pseudo-count, and applying a log-transformation. The order of bars is the same in (A) and (B).

**Fig. S2. Differentially abundant bacterial genera in individuals with no/slight and substantial tongue coating with a high fungal load.**

(A) Differentially abundant bacterial genera were identified between individuals with no/slight and substantial tongue coating and a high fungal load using MaAsLin2 analysis. Dots indicate genera with prevalence of ≥ 10%; the x-axis shows the effect size (coefficient), and the y-axis represents −log10 (adjusted *P* value). Threshold: adjusted *P* < 0.25. Negative values indicate genera that were more abundant in individuals with a no/slight tongue coating (pink), and positive values indicate genera that were more abundant in individuals with substantial tongue coating (dark gray). (B) Differentially abundant genera between individuals with no/slight and substantial tongue coating. Bar plots show effect sizes from MaAsLin2 for each species. Species with *q* value <0.25 are shown.

**Fig. S3 Relative abundance and detection ratio of differentially abundant bacterial taxa between individuals with no/slight and substantial tongue coating with a high fungal load.**

Bacterial species with differential abundances in individuals with no/slight and substantial tongue coating and a high fungal load are listed in descending order of the effect size from MaAsLin2. Species with *q* value <0.25 and an absolute effect size >1.0 are shown. Human Microbial Taxon (HMT) numbers from the expanded Human Oral Microbiome Database (eHOMD) are provided in parentheses following bacterial names. Species without HMT numbers were assigned based on the SILVA database.

**Fig. S4. Representative tongue coating images of individuals with no/slight tongue coating and a low fungal load.**

**Fig. S5. Principal coordinate analysis (PCoA) of bacterial composition among individuals classified by tongue coating status and fungal load.**

The points corresponding to individuals in each group are depicted in different colors. Ellipses accounted for 67% of the samples in each group. Statistical significance was calculated using PERMANOVA. No/slight TC and LF, no/slight tongue coating and a low fungal load (light pink); substantial TC and LF, a substantial tongue coating and a low fungal load (light gray); no/slight TC and HF, no/slight tongue coating and a high fungal load (pink); substantial TC and HF, a substantial tongue coating and a high fungal load (dark gray).

**Fig. S6. Relative abundances of seven discriminant bacterial species identified in Figure 5 in individuals with no/slight tongue coating and a high fungal load and in the other three groups.**

Relative abundances of seven discriminant bacterial species identified in Figure 5 are shown. Statistical significance was assessed using Dunnett’s test against the no/slight TC and HF group for CLR-transformed abundance. ^*^*P* < 0.05, ^**^*P* < 0.001, ^***^*P* < 0.001. No/slight TC and LF, no/slight tongue coating and a low fungal load (light pink); substantial TC and LF, a substantial tongue coating and a low fungal load (light gray); no/slight TC and HF, no/slight tongue coating and a high fungal load (pink); substantial TC and HF, a substantial tongue coating and a high fungal load (dark gray).

**Table S1. Characteristics of individuals classified according to tongue coating status and fungal load.**

| Characteristics | Substantial TC and LF  (n = 71) | No/slight TC and LF  (n = 16) | Substantial TC and HF  (n = 40) | No/slight TC and HF  (n = 18) | Significant differences between profiles  (*q* < 0.05) |
| --- | --- | --- | --- | --- | --- |
| Age (mean ± SD) | 86.5 ± 7.4 | 89.2 ± 4.7 | 88.3 ± 5.4 | 89.3 ± 6.4 | NS |
| Female (n, %) | 47 (66.2) | 14 (87.5) | 28 (70.0) | 13 (72.2) | NS |
| BMI (mean ± SD) | 23.0 ± 4.0 | 20.8 ± 3.8 | 22.0 ± 4.0 | 21.7 ± 3.9 | NS |
| Barthel index (mean ± SD) | 81.2 ± 20.5 | 85.0 ± 20.6 | 85.6 ± 14.1 | 77.1 ± 15.9 | NS |
| MMSE (mean ± SD) | 20.9 ± 5.4 | 20.7 ± 6.0 | 21.8 ± 5.2 | 18.8 ± 6.4 | NS |
| Number of present teeth (mean ± SD) | 10.2 ± 10.0 | 10.9 ± 9.4 | 11.2 ± 8.7 | 11.6 ± 8.8 | NS |
| Edentulous (n, %) | 25 (35.2) | 4 (25.0) | 5 (12.5) | 4 (22.2) | NS |
| Dentulous with ≥3 decayed teeth (n, %) | 18 (25.4) | 6 (37.5) | 12 (34.3) | 10 (71.4) | NS |
| Numbers of decayed teeth (mean ± SD) | 2.3 ± 2.5 | 3.8 ± 3.5 | 2.3 ± 3.6 | 6.6 ± 5.1 | substantial TC and HF < no/slight TC and HF, substantial TC and LF < no/slight TC and HF |
| Probing pocket depth (mean ± SD) | 3.2 ± 1.1 | 3.2 ± 0.4 | 3.1 ± 1.0 | 3.3 ± 0.5 | NS |
| Plaque score (mean ± SD) | 1.5 ± 0.8 | 1.6 ± 1.2 | 1.7 ± 0.9 | 2.0 ± 0.8 | NS |
| Percentage of teeth with bleeding on probing (mean ± SD) | 19.4 ± 25 | 23.5 ± 24.2 | 25.4 ± 30.4 | 25.2 ± 21.8 | NS |
| Denture users (n, %) | 53 (74.6) | 14 (87.5) | 32 (80.0) | 15 (83.3) | NS |
| Low oral moisture (n, %) | 17 (23.9) | 6 (37.5) | 12 (30.0) | 6 (33.3) | NS |
| Oral moisture (mean ± SD) | 10.8 ± 4.6 | 9.2 ± 6.1 | 10.5 ± 4.7 | 10.6 ± 3.6 | NS |
| Regular dental check-up (n, %) | 5 (7.0) | 1 (6.3) | 6 (15.0) | 2 (11.1) | NS |

SD, standard deviation. Pairwise comparisons were performed using the Wilcoxon rank-sum and Fisher’s exact tests. A *P* < 0.05 after Benjamini–Hochberg adjustment (*q* value) was considered statistically significant. NS indicates lack of statistical significance. Of these 145 individuals, 38 edentulous elderly individuals were excluded based on the number of decayed teeth, mean probing pocket depth, mean plaque score, and percentage of teeth with bleeding on probing. No/slight TC and LF, no/slight tongue coating and a low fungal load; substantial TC and LF, a substantial tongue coating and a low fungal load; no/slight TC and HF, no/slight tongue coating and a high fungal load; substantial TC and HF, a substantial tongue coating and a high fungal load.
